# Cross-Cultural Adaptation of Persian Version of Scale of Oral Health Outcomes for 5-Year-Old Children

**Published:** 2017-01

**Authors:** Imaneh Asgari, Elaheh Kazemi

**Affiliations:** 1 Assistant Professor, Dental Material Research Center, Department of Oral Public Health, School of Dentistry, Isfahan University of Medical Sciences, Isfahan, Iran; 2 Private Practice, Isfahan, Iran

**Keywords:** Quality of Life, Oral Health, Child, Surveys and Questionnaires

## Abstract

**Objectives:**

Indicators of oral health-related quality of life (OHRQoL) in children are widely adopted to evaluate the effects of oral problems. Recently, the scale of oral health outcomes for 5-year-old children (SOHO-5) was developed based on the children’s self-reports. This study aimed to evaluate the validity and reliability of the Persian version of the questionnaire in a sample of Iranian children.

**Materials and Methods:**

This cross-sectional study was conducted on 160 children from four areas of Isfahan selected via non-random purposive sampling. After forward-backward translation of the questionnaire, content and face validity evaluation, a pilot test was carried out. Children forms were completed by interview, while parents forms were self-administered. Test-retest reliability was evaluated in 30 subjects. Construct validity, internal consistency and descriptive quality of life score were assessed with SPSS 18. The child-parent agreement was measured with correlation test and paired t-test (α=0.05).

**Results:**

The mean (±standard deviation) quality of life scores in children and parents were 2.3±3 and 1.3±1.9, respectively. The most prevalent impacts were difficulty sleeping and eating. The Cronbach’s alpha coefficients were 0.82 and 0.67 for the child and parent versions, respectively. Significant correlation of the scores with the oral health rating, pain history and perceived need for treatment confirmed its construct validity (r: 0.4–0.6, P<0.05). The hypothesis of the agreement was not supported (P>0.05).

**Conclusions:**

Based on the findings, the Persian version of SOHO-5 has acceptable reliability and validity for use in the pediatric population of Iran while there were some conflicts by parents.

## INTRODUCTION

By expanding the concept of oral health-related quality of life (OHRQoL) within the scope of outcome measures, individual self-reports have been emphasized in addition to traditional disease diagnosis [1]. Oral health related quality of life evaluates impacts of oral pain and dysfunction on psychological and social performance. Various tools have been designed to measure the qualitative experiences and relationship between oral problems and personal and social life. Children’s oral health affects their feeding and social relationship as well as speaking and smiling [2]. Despite the improvement of oral health, dental caries is a public problem all around the world and also in Iran. The mean dmft of 4.13 in 3–6 year-olds and 61% prevalence of childhood caries have been reported in different areas of Iran [3,4]. The oral health national survey in 2012 showed that only 12.7% of primary teeth were caries-free among 5–6 year-old children [5]. Therefore, preschool children are still among the prioritized target groups for oral health policies. According to children’s psychology, changes occur in intelligence, language development, speaking, ability to communicate, imagination and logical thinking related to the concepts such as health and disease [6]. Therefore, various measuring tools have been designed from pre-school to adolescence in the field of OHRQoL.

In young children, quality of life status is measured by parents’ report because of the concerns about psychological standards, validity and consistency of their answers [7–11]. Evidence shows that abstractive intelligence such as well-being issues in 4-6-year-old children cannot be reliable; however, other literatures supported that tangible issues of health such as pain and dysfunction could be reported by children consistently [12].

Recently, with a qualitative and quantitative method, the scale of oral health outcomes for 5-year-olds (SOHO-5) was developed and assessed for the reliability and validity in the United Kingdom. It was the unique self-reported instrument in the field of measuring the impacts of oral health in children. As it provides both child self- and proxy reports, this instrument can be interesting for professionals involved in oral health services and research [13]. It was also validated in Brazilian Portuguese after cross-cultural adaptation [14]. Based on limited information about OHRQoL in the Iranian children, the aim of this study was to evaluate the validity of Persian version of SOHO-5 among children living in Isfahan. Moreover, we assessed the parent–child agreement on rating children’s OHRQoL.

## MATERIALS AND METHODS

The study protocol was approved in the ethics committee of Isfahan University of Medical Sciences (code:393714). By obtaining permission from questionnaire developer, children and parents forms, in addition to the prompt response card were translated by two bilingual translators, independently, to Persian in accord with the standard guidelines. The back-translated version was compared with the original forms to determine the semantic equivalence. After reaching a consensus, a single copy was prepared by the translation team and researchers.

In terms of face and content validity, the translated version was evaluated by a panel of experts including pedodontists, public health dentists and child psychologists. According to Lynn’s method, relevance of each question was measured with a scale of 1: completely irrelevant to 4: completely relevant [15]. Content validity index for each item was calculated by the proportion of items with the scores 3 or 4 and the cut-off point of 67% by experts. Overall, scale-content validity index (S-CVI) was reported by both universal agreement (ua) formula and overall scale CVI by average (ave) [16]. Considering the final changes, the last draft of Persian version was pilot tested in 15 children in the age of 5–6 years for comprehensibility of questions, response time and the way of communicating with children. Consequently, the psychometric properties of Persian SOHO-5 were assessed in a sample of target group.

Based on previous standard deviation of 3.2 [14], α equal to 95% and measurable error of 0.5, a sample of 160 children in the age of 5–6 years were recruited for the descriptive study from four kindergartens (approximately 40 from each kindergarten) of different social regions of Isfahan city.

They were selected randomly according to the high (areas 3 and 5) and low socioeconomic class (areas 10 and 12) municipal subdivisions. Written informed consent was obtained from children’s parents or guardians. The children form was filled out by a face-to-face independent interview by asking children about pain experience and the effects of oral status on their daily activities. Children were queried about any experience of difficulty in eating, drinking, smiling (due to dental caries and appearance) and sleeping. The answers were recorded using a 3-point scale (no: 0, a little: 1 and a lot: 2). Parents forms were given to parents and collected after one week. The self-administered questionnaire was about their child’s difficulties in eating, playing, speaking, sleeping and avoiding smiling due to tooth decay and unesthetic appearance of teeth negatively affecting their self-confidence. Response options were in the form of a Likert scale: Never: 0, a little:1, moderate: 2, a lot: 3, a great deal: 4. The SOHO-5 scores were calculated as the sum of response scores for each questionnaire. This form had three parts including current toothache in children, the impact of child’s teeth on daily life (seven items) and the effects of dental health on the family (11 items). After collecting data, similarity between parents’ and children’s responses about evaluation of the impact of oral problems on quality of life was assessed. For this purpose, six common questions in two forms were selected and the two questionnaires was re-coded in a three-point scale (never=0, a little and moderate=1, a lot and a great deal=2). Therefore, the score for the evaluation of both forms was calculated from 0 to 12. To assess the construct validity of the questionnaire, standard global rating questions were asked. For the children, satisfaction with oral health (not happy=2, a little happy=1 and very happy=0) and presence of dental cavities (no=0, yes=1) were questioned. For the parental questionnaires, the following oral health ratings (excellent=0, very good=1, good=2, fair=3, poor=4), satisfaction with child’s oral health (very happy=0 to very unhappy=4), and the child’s perceived dental treatment needs (no=0, yes=1) were included. Subjective dental health questions as current and experience of toothache were asked for testing discriminant validity. *Data analysis:* The collected data were analyzed using SPSS software version 18. The final overall scores ranged from 0 to 14 for the child and from 0 to 28 for the parents version with seven questions. The score of impacts on the family was reported in the range of 0 to 44 for the final 11 items. Answers of “I cannot remember” or “I do not know” were considered as missing data. For the self-administered questionnaire, missing data were replaced by nearby points’ median to calculate the total scores. Quality of life mean (standard deviation) scores from children and parents forms and the frequency of each dental problem were reported as descriptive statistics. Moreover, the test-retest reliability of SOHO child form was calculated after it was completed by 30 children via an interview in a two-week interval. Internal consistency of the questionnaire was assessed by Cronbach’s alpha coefficient and the inter-item correlation. Construct convergent validity was tested through associations between the SOHO-5 scores and the global ratings using Spearman’s correlation coefficients. Comparing the means between the children with current/history of toothache, cavity or perceived need to treatment was done for discriminant validity. The agreement between the two forms was examined by intraclass correlation coefficient (ICC).Degree of agreement was classified as poor (<0.2), fair (0.2–0.4), moderate (0.41–0.6), substantial (0.61–0.8) and excellent (0.81–1) [17].

## RESULTS

Content validity index for items of children form indicated that all the statements with exception of discomfort in tooth eruption (Q1) had a mean index of more than 3 with 83% acceptable response rate. In children form, the S-CVI was 0.5 (ua) and 0.89 (ave). Hence, the first question was omitted and the final questionnaire was changed to six items with a response rate of zero to 12. In the parents form, question 22 about being jealous of family member by 66% acceptable CVI response rate and question 24 about blaming the parents by child were omitted. In parents form, the S-CVI was 0.62 (ua) and 0.96 (ave). The questions 21 and 23 were merged due to content similarity. The pilot test showed that children preferred to express their feelings or experiences verbally rather than pointing to the cards with schematic pictures, and average response time was seven minutes for each interview. Almost all the subjects answered without requiring repetition or rewording. A total of 82 boys (51%) and 78 girls enrolled in the main study, and the mean of quality of life score was found to be 2.3±3 with a range of 0–14 in children form. The most common problems were difficulty in sleeping (32%) and eating (33%), while the least common problem was avoiding smiling due to tooth decay (11%). Approximately, 90% of the parents questionnaire was answered (response rate) and the parental SOHO score ranged from 0–9 with a mean of 1.3±1.9. The score of the family impact section with 84% response rate showed a mean of 8.3±7 with a range of 0–26. Nearly, 41% of the children and 46% of the parents reported no oral impacts of daily living but 88% of them had encountered problem in their family. Children’s SOHO mean score was significantly higher in boys than girls (P=0.027), although this relationship was not significant in parents form. However, the parents’ score of quality of life was more in lower socioeconomic regions than in upscale areas (P<0.001) but this was not significant in case of children. In terms of internal consistency reliability, Cronbach’s alpha coefficient was 0.82 and 0.67 in children and parents’ questionnaires, respectively (Tables 1 and 2). To evaluate the reliability over time, test-retest showed ICC (95% confidence interval) of 0.8 (range 0.6–0.9) for parents form. The two-way random effect model analysis showed a P<0.001. In addition, the mean difference test (paired sample t-test) at two times showed no significant difference (P=0.6) over time. The construct convergent validity showed that the SOHO-5 total score was associated significantly and in the expected direction with two global rating questions for parental proxy reports (Table 3). Eating and sleeping items were significant subscales with moderate correlation. This was not supported in the child version when the relation between the question about “satisfaction with oral health” and total score was analyzed (P>0.05). The discriminant validity was clarified by significant mean differences between the scores of quality of life regarding three subjective questions about toothache and cavities. For both versions, children with a history of toothache or cavities in their teeth exhibited significantly higher SOHO-5 total scores compared to children with no history of caries (Tables 4 and 5).

**Table 1:** Internal consistency reliability of SOHO-5 subdomains of children forms

	**Corrected item-total correlation coefficients**	**Alpha if item deleted**
Difficulty eating	0.80	0.50
Difficulty drinking	0.78	0.63
Difficulty speaking	0.77	0.68
Difficulty playing	0.78	0.65
Avoiding smiling (due to pain)	0.80	0.50
Avoiding smiling (due to appearance)	0.79	0.58
Difficulty sleeping	0.82	0.45

**Table 2:** Internal consistency reliability of SOHO-5 subdomains of parent forms

	**Corrected item-total correlation coefficients**	**Alpha if item deleted**
Difficulty eating	0.56	0.44
Difficulty speaking	0.57	0.33
Difficulty playing	0.56	0.37
Avoiding smiling (due to appearance)	0.61	0.13
Avoiding smiling (due to pain)	0.6	0.25
Difficulty sleeping	0.44	0.62
Affected self-confidence	0.6	0.25

**Table 3:** Construct validity for the SOHO-5 parent forms using binary correlation test

	**Proxy-rated oral health**		**Satisfaction with child’s oral health**	

**Questionnaire subdomains**	r^*^	**P-value**	r^*^	**P-value**
Total score	0.5	0.00	0.4	0.00
Difficulty eating	0.4	0.00	0.4	0.00
Difficulty speaking	0.13	0.10	0.15	0.70
Difficulty playing	0.1	0.10	0.2	0.01
Avoiding smiling (due to appearance)	0.1	0.10	0.005	0.95
Avoiding smiling (due to pain)	0.1	0.20	0.7	0.37
Difficulty sleeping	0.4	0.00	0.42	0.00
Affected self-confidence	0.16	0.04	0.15	0.05

* Spearman’s correlation coefficient

**Table 4:** Discriminant validity of children forms of SOHO-5 with subjective clinical oral health indicators

	**N**		**Mean**	**P-value**
Toothache (current)	Yes	126	4.8	0.0001
	No	34	1.6	
Toothache (experience)	Yes	61	4.1	0.0001
	No	99	1.1	
Reported cavities	Yes	41	4.1	0.0001
	No	117	1.6	

**Table 5:** Discriminant validity of parents form of SOHO-5 with subjective oral health indicators

	**N**		**Mean**	**P-value**
Perceived need for treatment of parents	Yes	71	2.2	<0.0001
	No	55	0.38	
Toothache (experience)	Yes	78	2.2	<0.0001
	No	65	0.35	
Toothache (current)	Yes	45	2.7	<0.0001
	No	98	0.72	

The findings of agreement analysis between six common items in children and parents forms demonstrated that in all items, correlation between children and parents responses was at the poor or fairly poor extent. The highest reached agreement was at the domain of impacts on sleeping (ICC of 0.28, P=0.02). The mean overall quality of life score in the common items was 2.5±1.8 and 1.2±1 for children and parents, respectively, and showed a significantly lower score for parents compared with children (P=0.001, paired t-test analysis).

## DISCUSSION

This study was designed with the aim of preparing and validating the SOHO-5 Persian version in 5-year-old Iranian children living in Isfahan.

The quality of life questionnaire specialized for this range of age was surveyed in terms of face, content, and construct validity. In this study, a self-reported OHRQoL measuring tool for children was prepared for the first time in Persian language.

The psychometric properties of the SOHO-5 were satisfactory and provided strong support for its reliability and validity. The range of inter-item correlation coefficient was 0.15–0.73, which was above the recommended level of 0.2. Cronbach’s alpha was 0.82 and it was lower when any of the items was deleted. In the original English version by Tsakos et al, [13] this range was 0.11–0.44 and Cronbach’s alpha was 0.74. While the value of alpha tends to be higher for questionnaires with more questions, our study revealed very good internal consistency for the SOHO-5 with limited queries. The results of test-retest reliability with ICC of 0.8 was a little less than that in the study by Abanto et al, [14] in Brazilian version with the ICC of 0.92. The measure also demonstrated discriminant validity between clinical groups according to their subjective caries history and perceived treatment need. However, the ability of this tool for discrimination between clinical conditions would be proven after standard clinical examination in future studies.

Quality of life scores of children version with a mean of 2.3±3 was more than the English population in the study by Tsakos et al, [13] with a mean of 1.38±3 and less than Brazilian children in the study by Abanto et al, [18] with a mean of 3.32±3.2. However, the reason for higher score in their study can be selecting the target population from children who had decay and trauma histories. The most common difficulties were eating and sleeping and the least prevalent problem was avoiding smiling due to decay in both Iranian and British children [13]. In a similar way, Abanto et al, [14] showed difficulties with eating and sleeping and avoiding smiling due to appearance as the most common problems. Obviously, the questions on the items “avoid smiling due to appearance and decay” need more explanation or repeating for children as in the study on British children [13].

In our study, the parents’ score of SOHO was in the range of 0–9 with a mean of 1.3±1.9, although, in the studies by Abanto et al, [14,18] this range was broader (0–24) with a higher mean (3.67±5.54). The observed dissimilarities between the results may be caused by different perceptions of the quality of life subdomains, which are affected by culture, education, and other social conditions in the communities. Furthermore, sampling among general population or volunteer patients for treatment affects the results.

Global health rating and perceived oral health or satisfaction with teeth are the general indices that are implicated for testing the convergent construct validity. In our study, the questions which have been utilized to evaluate construct validity in parents version showed good result, in spite of the non-significant answers due to the children’s happiness with their teeth. Difficulty in eating and sleeping were the most relevant problems with general oral health perception and satisfaction in our study. Moreover, the correlation between child’s toothache with difficulties in eating, sleeping and avoiding smiling due to appearance was similar to the results of the Brazilian study [14]. The highly expressed eating and sleeping subdomains in our study supported understanding of more tangible terms. Recently, we encountered a rapid change in the structure of personal and family life because of digital technology. With the rapid advancements in the devices like smartphones or digital tablets, there is an explosion of electronic media and learning packages directed at preschool children in many societies. It is stated that both positive and negative impacts on children’s development would be seen in physical, cognitive and social domains [19]. Thus, constructing subjective measures for young children would be challenging in future. To date, nearly most of the research on OHRQoL measures has established on the parents’ proxy reports. While the parents’ expression may not necessarily be identical to those of their children, the relevant studies have shown weak or moderate associations between them [7,18, 20]. Although another study on SOHO-5 revealed that parents reported significantly worse OHRQoL than their children [18], our results showed overall better quality of life statements in parents compared with their children’s reports while the agreement was only in the difficulty in sleeping. Therefore, the newly introduced tool for measuring the quality of life by children reports should be evaluated in large or diverse samples and in symptomatic patients to clarify the real competencies. However, it is controversial whether there is an accurate patient-based tool compatible with clinical manifestations in children, and the statistical validity and reliability of the instrument may not guarantee the fact. Discriminant construct validity of both children and parents forms of the questionnaire should be tested in future studies.

## CONCLUSION

According to the results of this study, Persian version of SOHO-5 is acceptable for 5-year-old Persian language children as a complementary assessment measure while it could not be a substitute tool.

## References

[1-] Slade DG Measuring oral health and quality of life. University of North Carolina at Chapel Hill. Department of Dental Ecology, School of Dentistry, University of North Carolina; 1997:160–4.

[2-] Sheiham A Tsakos G Oral health needs assessment In Pine CM Harris R Community Oral Health. Second ed., Edinburgh, Quintessence Publishing Co., 2007:40–56.

[3-] Edalat A Abbaszadeh M Eesvandi M Heidari A The relationship of severe rarly childhood caries and body mass index in a group of 3- to 6-year-old children in Shiraz. J Dent (Shiraz). 2014 6;15(2):68–73. 24883343 PMC4033086

[4-] Toutouni H Nokhostin MR Amaechi BT Zafarmand AH The prevalence of early childhood caries among 24 to 36 months old children of Iran: Using the novel ICDAS-II method. J Dent (Shiraz). 2015 12;16(4):362–70. 26636126 PMC4664035

[5-] Mazhari F Talebi M Zoghi M Prevalence of Early childhood caries and its risk factors in 6–60 months old children in Quchan. Dent Res J. 2008 7;4(2):96–101.

[6-] Parke RD Gauvain M Child psychology: A contemporary viewpoint. 7th Ed., Boston, MA, McGraw-Hill, 2009:153–6.

[7-] Barbosa TS Gavião MB Oral health-related quality of life in children: part III. Is there agreement between parents in rating their children’s oral health-related quality of life? A systematic review. Int J Dent Hyg. 2008 5;6(2):108–13. 18412722 10.1111/j.1601-5037.2007.00271.x

[8-] Nurelhuda NM Ahmed MF Trovik TA Åstrøm AN Evaluation of oral health-related quality of life among Sudanese schoolchildren using Child-OIDP inventory. Health Qual Life Outcomes. 2010 12;8:152. 21182769 10.1186/1477-7525-8-152 PMC3019139

[9-] Asgari I Ahmady AE Broder H Eslamipour F Wilson-Genderson M Assessing the oral health-related quality of life in Iranian adolescents: validity of the Persian version of the Child Oral Health Impact Profile (COHIP). Oral Health Prev Dent. 2013;11(2):147–54. 23534043 10.3290/j.ohpd.a29367

[10-] Pahel BT Rozier RG Slade GD Parental perceptions of children’s oral health: the Early Childhood Oral Health Impact Scale (ECOHIS). Health Qual Life Outcomes. 2007 1;5:6. 17263880 10.1186/1477-7525-5-6 PMC1802739

[11-] Foster Page LA Boyd D Thomson WM Do we need more than one Child Perceptions Questionnaire for children and adolescents. BMC Oral Health. 2013 6;13:26. 23758709 10.1186/1472-6831-13-26 PMC3686591

[12-] Connolly MA Johnson JA Measuring quality of life in pediatric patients. Pharmacoeconomics. 1999 12;16(6):605–25. 10724790 10.2165/00019053-199916060-00002

[13-] Tsakos G Blair YI Yusuf H Wright W Watt RG Macpherson LM Developing a new self-reported scale of oral health outcomes for 5-year-old children (SOHO-5). Health Qual Life Outcomes. 2012 6;10:62. 22676710 10.1186/1477-7525-10-62 PMC3413607

[14-] Abanto J Tsakos G Paiva SM Goursand D Raggio DP Bönecker M Cross-cultural adaptation and psychometric properties of the Brazilian version of the scale of oral health outcomes for 5-year-old children. Health Qual Life Outcomes. 2013 2;11(1):16. 23394292 10.1186/1477-7525-11-16 PMC3600026

[15-] Lynn MR Determination and quantification of content validity. Nurs Res. 1986 Nov-Dec;35(6):382–5. 3640358

[16-] Polit DF Beck CT The content validity index: are you sure you know what’s being reported? Critique and recommendations. Res Nurs Health. 2006 10;29(5):489–97. 16977646 10.1002/nur.20147

[17-] Landis JR Koch G The measurement of observer agreement for categorical data. Biometrics. 1977 3;33(1):159–74. 843571

[18-] Abanto J Tsakos G Paiva SM Carvalho TS Raggio DP Bönecker M Impact of dental caries and trauma on quality of life among 5- to 6-year-old children: perceptions of parents and children. Community Dent Oral Epidemiol. 2014 10;42(5):385–94. 24460685 10.1111/cdoe.12099

[19-] Wu CS Fowler C Lam WY Wong HT Wong CH Yuen Loke A Parenting approaches and digital technology use of preschool age children in a Chinese community. Ital J Pediatr. 2014 5;40:44. 24887105 10.1186/1824-7288-40-44 PMC4046626

[20-] Jokovic A Locker D Guyatt G How well do parents know their children? Implications for proxy reporting of child health-related quality of life. Qual Life Res. 2004 9;13(7):1297–307. 15473508 10.1023/B:QURE.0000037480.65972.eb

